# Outcome analysis of immediate and delayed laparoscopic pyeloplasty in infants with severe ureteropelvic junction obstruction

**DOI:** 10.3389/fped.2022.1022836

**Published:** 2022-10-19

**Authors:** Qiao Bao, Weijun Ma, Xiewu Zhang, Shuhan Chen, Jiayao Luo, Gang Zhang, Weihua Lao, Yueqing Chen

**Affiliations:** ^1^ Department of Pediatric Urology, Guangdong Women and Children Hospital, Guangzhou, China; ^2^ Department of Pediatric Surgery, The Third Affiliated Hospital of Guangzhou Medical University, Guangzhou, China

**Keywords:** infant, ureteropelvic junction obstruction, immediate, delayed, laparoscopic pyeloplasty

## Abstract

**Objective:**

The treatment timing of ureteropelvic junction obstruction (UPJO) in infants remains controversial. This study aimed to compare the recovery effect of renal morphology of immediate and delayed laparoscopic pyeloplasty in infants with severe UPJO.

**Methods:**

The infants with severe UPJO-induced hydronephrosis who underwent laparoscopic pyeloplasty according to their age at the time of surgery [the immediate treatment (IT) group: ≤1 month of birth, the delayed treatment (LT) group: 3–6 months of birth] in our center between 2010 and 2019 were enrolled in this study. Ultrasonography was used to assess renal morphology, including anteroposterior diameter (APD) of a pelvic, parenchymal thickness (PT), polar length (PL), and Society of Fetal Urology (SFU) grade. Preoperative and postoperative renal morphological outcomes at 6, 12, and 24 months were measured and compared.

**Results:**

During this period, a total of 135 patients were assigned to receive either IT (*n* = 73) or LT (*n* = 62) and were included for analysis. There were no significant differences in renal morphology indices at baseline between groups of IT and LT. The APD, PT, and PL in both groups all recovered to certain degrees compared with those at baseline, however, the IT group recovered more significantly than the LT group. Despite there being no significant difference in SFU grade between the two groups before and after surgery, the reduction of SFU grade in the IT group was more significant than that in the LT group during the 6-, 12- and 24-month follow-up periods. The PL, SFU, and APD were greater in the IT group than in the LT group at 6, 12, and 24 months of follow-up. At 6 months PL was not significantly higher between the two groups, while the outcome was significantly different at 12 months and 24 months.

**Conclusion:**

Immediate laparoscopic pyeloplasty for the infant with severe ureteropelvic junction obstruction is effective, and it can accelerate the recovery of renal morphological indices in infants with severe UPJO-induced hydronephrosis.

## Introduction

Ureteropelvic junction obstruction (UPJO) is the most common cause of prenatal and neonatal hydronephrosis (1). The prevalence rate is estimated to be 1 in 1500 live births, with a male to female ratio of 3–4 to 1 (2). With the prevalence of fetal screening ultrasonography, the detection of UPJO enables the management of the condition (3, 4). Therefore, how to effectively treat infant hydronephrosis caused by UPJO has become a major concern in pediatric urological practice.

Laparoscopic pyeloplasty is an effective surgical treatment for the recovery of the function and morphology of the UPJO-affected kidney (5). Some current literature suggested that pyeloplasty facilitated the improvement of anatomic and functional indices, while conservative management might lead to a significant deterioration in renal function (6–8). However, other reports indicated that a large proportion of cases were stabilized or resolved following conservative management (9, 10). Thus, there is an urgent need for an appropriate regime that provides better treatment outcomes.

Since the obstruction leads to a decrease in urine flow from the renal pelvis into the ureter, the main mechanism that triggers renal parenchymal damage is increased pelvicalyceal system pressure, untreated UPJO may lead to chronic infection or urolithiasis, and often results in progressive deterioration of renal function (11). For this reason, we insist that prompt laparoscopic pyeloplasty can prevent permanent loss of renal function in infants with severe hydronephrosis. Nevertheless, the optimal time for surgical intervention in infants with severe UPJO remains controversial.

A previous long-term follow-up of children with hydronephrosis caused by UPJO who received surgical treatment within 1-year-old found that the incidence of postoperative reoperation and the risk of rehospitalization were higher than those who received surgery over 1 year old (12). They indicated that younger age at pyeloplasty was associated with a higher risk of readmission. However, some advocated that surgical treatment of UPJO in the early stage of life was safe and effective (13). Furthermore, several studies have shown that surgical intervention should be conducted as early as possible after severe hydronephrosis occurs in the kidney (14, 15). So, it is necessary to assess the clinical significance of age based on the time of surgical correction in these children.

Therefore, this retrospective cohort study aimed to compare the changes in renal morphology between infants receiving IT and those undergoing DT for severe hydronephrosis. The findings in this study might provide clinical guidance for the treatment of infants with severe UPJO.

## Material and methods

This is a retrospective cohort study of infants with prenatally diagnosed severe hydronephrosis with UPJO who were admitted to our department from 2010 to 2019. All cases of prenatal UPJO-induced hydronephrosis were diagnosed by screening ultrasonography. Detailed ultrasonography was performed within 7 days after birth to confirm the prenatal diagnosis of severe hydronephrosis again. Infants undergoing laparoscopic pyeloplasty within 1 month of birth formed the IT group, whereas those undergoing laparoscopic pyeloplasty at 3–6 months of birth formed the DT group. The inclusion criteria were lack of obvious clinical symptoms and severe hydronephrosis as defined by SFU grade 3 or 4 (16). Infants with bilateral hydronephrosis, obvious clinical symptoms, less than a 24-month follow-up period, or other urinary malformations were excluded from this study. Renal morphological evaluation was performed using ultrasonography examination at 6, 12 and 24 months after surgery. Age, gender, side of UPJO, preoperative and postoperative renal indices, and data on the follow-up were recorded.

The condition of each patient was described to their parents, the acceptability of the two treatment methods was clarified, and the possible related advantages and disadvantages of each strategy were discussed. Then, according to the actual situation of the patients, together with their parents, decide whether to conduct immediate or delayed laparoscopic pyeloplasty and sign the informed consent form. The study was performed following the principles of the Declaration of Helsinki.

All enrolled infants received a standard laparoscopic pyeloplasty procedure by the experienced and same surgical team at our department. Before surgery, full intestinal preparation was made, and anal canal could be placed if necessary to increase the operation space and better expose the surgical field of vision. Classical laparoscopic Anderson-Hynes dismembered pyeloplasty was done in all patients (17). First, a 5 mm trocar was placed for the camera at the site of the umbilicus. Pneumoperitoneum was created and two 3 mm trocars were placed more laterally in the upper and lower quadrants of the abdomen. For the left side cases, the transmesenteric approach was adopted, whereas for right side cases, we selected the paracolic sulci approach. Then we carefully dissected the proximal ureter and renal pelvis to prevent ureteral blood damage. The pelvis was cut above the obstruction tissue and carefully trimmed to facilitate the anastomosis. Thereafter, a 5-0 absorbable was used to suture the lowest point of the ureteral segment and the pelvis end (18). Before the anterior anastomoses were started, a properly sized indwelling double- J stent (Bard, U.S.) was inserted and prophylactic antibiotics were initiated until the double-J stent was removed on the 6–8th week after surgery.

The data were processed using IBM SPSS statistical software version 20 (IBM, Armonk, NY, USA). Descriptive statistics were used and results were expressed as medians with interquartile ranges (IQRs). The chi-square test and the t-test were adopted to compare measurement and count data, respectively. Statistical significance was considered at *P* < 0.05.

## Results

Over the 2-year follow-up of the study, a total of 162 patients met the inclusion criteria. Among them, 27 were excluded owing to presence of clinical symptoms (*n* = 9), less than 9 months follow-up period (*n* = 7), bilateral disease (*n* = 6), or other urinary malformations (*n* = 5). Eventually, the remaining 135 infants were divided into the IT group (*n* = 73) and the DT group (*n* = 62).

The initial characteristics of these 135 infants are shown in Table 1. All infants with prenatal hydronephrosis had ultrasonography confirmation of UPJO within 7 days of birth. Of the patients, 86% of the children were boys, and 79% of UPJO occurred on the left lateral side. There were no significant differences in the general morphological characteristics between the two groups. All included infants were classified as having severe obstruction with a baseline SFU grade of 3 or 4 (Table 1).

**Table 1:** Initial characteristics of hydronephrosis in infants.

Variable	IT group (73)	DT group (62)	*p*-Value
Age at time of inclusion	Within 7 days of birth	Within 7 days of birth	
Gender			0.892
Male	63 (86%)	53 (85%)	
Female	10 (14%)	9 (15%)	
Laterality			0.802
Left	56 (77%)	50 (81%)	
Right	17 (23%)	12 (19%)	
APD of the renal pelvis (cm)	2.22 (1.91–2.51)	2.24 (2.02–2.48)	0.589
SFU Grade 3/4	41/32	32/30	0.600
PT (cm)	1.90 (1.60–2.30)	1.95 (1.68–2.20)	0.797
PL (cm)	4.00 (3.45–4.80)	4.20 (3.08–5.02)	0.613

Values are expressed as median with interquartile ranges (IQR) or *n* (%).

IT, immediate treatment; DT, delayed treatment; APD, anteroposterior diameter; PT, parenchymal thickness; PL, polar length; SFU, Society of Fetal Urology.

Preoperative and postoperative comparisons of renal morphology between the two groups are shown in Table 2. The APD of the renal pelvis decreased in both groups than before surgery, but the decrease was more significant in the immediate treatment group (*P* < 0.001). In the IT group, the initial median of PT and PL of renal improved from 1.90 to 2.15 and 1.95 to 1.99 at the 6-month follow-up after surgery, respectively. Compared with the DT group, the size of the kidney in the IT group increased more significantly (both *P* < 0.001). Moreover, the IT group also had a higher SFU grade decrease than the DT group, but the difference was not significant (*P* = 0.058).

**Table 2:** Preoperative and postoperative changes in renal morphology.

Variable	IT Group	DT group	*p*-Value
The difference in APD of the renal pelvis (cm)	0.40 (0.30–0.43)	0.24 (0.18–0.31)	<0.001
The difference in PT (cm)	0.24 (0.21–0.30)	0.15 (0.09–0.23)	<0.001
The difference in PL (cm)	0.60 (0.50–0.72)	0.43 (0.34–0.59)	<0.001
Difference in SFU	0.52 (0.31–0.59)	0.33 (0.25–0.41)	0.058

Values are expressed as median with interquartile ranges (IQR).

IT, immediate treatment; DT, delayed treatment; APD, anteroposterior diameter; PT, parenchymal thickness; PL, polar length; SFU, Society of Fetal Urology.

During the 6-, 12- and 24-month follow-up periods, the APD of the renal pelvis was decreased in both groups. More significant changes in the APD of the renal pelvis were observed in the IT group than in the DT group (both *P* < 0.001) (Table 3). At the 6-month follow-up, the PL was improved in both groups, but there was no significant difference between the two groups (*P* = 0.284). However, at 12 and 24 months, there was a significant recovery in the IT group than in the DT group (*P* = 0.043). In addition, the IT group also achieved a faster recovery of PT and a greater SFU grade compared with those in the DT group at 6, 12, and 24 months (both *P* < 0.05) (Table 3).

**Table 3:** Follow-up characteristics of hydronephrosis in infants.

Variable	Follow-up (months)	IT Group	DT group	*p-*Value
APD of the renal pelvis (cm)	6	1.86 (1.60–2.10)	2.11 (1.90–2.34)	<0.001
	12	1.31 (1.12–1.47)	1.52 (1.37–1.68)	<0.001
	24	0.99 (0.95–1.21)	1.15 (1.11–1.21)	<0.001
PT (cm)	6	2.15 (1.81–2.60)	1.99 (1.71–2.24)	0.018
	12	2.47 (2.08–2.99)	2.18 (1.88–2.46)	<0.001
	24	3.00 (2.84–3.13)	2.67 (2.24–2.89)	<0.001
PL (cm)	6	4.60 (3.97–5.52)	4.54 (3.32–5.43)	0.284
	12	5.40 (4.66–6.48)	5.12 (3.75–6.13)	0.043
	24	6.25 (5.83–6.51)	5.66 (5.03–6.09)	<0.001
SFU	6	3 (3–3)	3 (3–3)	0.037
	12	2 (2–3)	3 (2–3)	0.002
	24	2 (1–2)	2 (2–3)	<0.001

Values are expressed as median with interquartile ranges (IQR).

IT, immediate treatment; DT, delayed treatment; APD, anteroposterior diameter; PT, parenchymal thickness; PL, polar length; SFU, Society of Fetal Urology.

## Discussion

Although many studies on infants with prenatally diagnosed severe hydronephrosis with UPJO have been reported recently, the management of infant UPJO is still complex and unified. Most controversially, whether these patients should be aggressively treated with surgery or conservatively observed. The main goal in the treatment of UPJO is to preserve or improve renal function (19, 20), we stressed no difference that conservative management of some patients with antenatally detected severe UPJO probably results in irreversible loss of function (21) and followed up with this study, which showed that immediate laparoscopic pyeloplasty accelerated the recovery of renal morphology in infants with severe UPJO-induced hydronephrosis. Immediate surgical intervention is effective and should be performed during this period.

Renal morphological indices, such as APD of pelvic and PT, are the most used measures to assess the severity of hydronephrosis and predict the improvement of renal function after pyeloplasty (22, 23). In our study, APD of pelvic and PT of infants in both groups recovered to varying degrees after surgery and during follow-up, but the IT group was more significant. In a recent retrospectively study, Li et al. assessed the effect of robotic-assisted laparoscopic pyeloplasty (RALP) performed in infants under 3 months old (18). Their experience also revealed that early RALP could significantly improve APD of pelvic for severe UPJO infants under 3 months. Recently, some studies have pointed to shortcomings of relying on APD of pelvic and have suggested that the degree of calyceal dilatation and PT may be more important (24). To overcome these shortcomings, more sophisticated grading systems have been described, such as the SFU grade system (25). Therefore, to ensure the reliability of the results of this study, we further evaluated the infants with severe hydronephrosis according to the SFU classification rather than APD or PT, and the results showed that the IT group also had a higher SFU grade decrease than DT group, but the difference was not significant. Satisfactorily, the reduction of SFU grade in the IT group was more significant than that in the DT group during the 6-, 12- and 24-month follow-up periods. We think it may be the reason that the patients are younger in our study, and they may need some time to recover after surgery. Another reason may be that SFU has an inferior resolution (26). All in all, the results showed that in high SFU grade UPJO infants, the immediate laparoscopic pyeloplasty could hasten the recovery of renal morphology.

Additionally, we evaluated renal PL change after pyeloplasty. The results demonstrated that at 6 months PL was not significantly higher between the two groups, while the outcome was significantly different at 12 and 24 months. This could be, because there is a large difference in kidney size between individuals at birth, and the affected degree of PL due to hydronephrosis detected by ultrasonography is not as intuitive as APD of pelvic and PT (26). Moreover, with increasing age and kidney development, this difference will become more significant. Taken together, these results once again demonstrated the positive impact of timely treatment in these patients with severe hydronephrosis.

The recovery of renal function after surgery is the main issue in the treatment of UPJO patients. The cornerstone of the vast majority of antenatal hydronephrosis is estimated differential renal function on dynamic renography (27, 28). However, in the present study, we did not perform radionuclide renogram measurements on these infants. On the one hand, our view is consistent with previous authors’ view that dysfunctional kidneys will try to compensate for the loss of renal function through glomerular hyperfiltration of the remaining tissue (29, 30) and/or by mobilizing at least part of their functional reserve (31). On the other hand, the kidneys mature at about 18 months, and the glomerular filtration function of immature kidneys is low, which often leads to poor image quality and inaccurate assessment during the calculation of differential renal function (DRF) using dynamic renal imaging (32, 33). As a result, we believe that the significance of this examination for these patients at such a young age is not so necessary, and it will not bias our research results.

Since this was a retrospective study, the population size and duration of follow-up were limited. In addition, since randomization was not ethically feasible, it was not completely random. Despite these shortcomings, our study showed several significant findings that confirm the significance of immediate pyeloplasty for the current management of infants with severe UPJO-induced hydronephrosis. A prospective and larger study should be conducted in detail to better assess the importance of immediate laparoscopic pyeloplasty in these patients.

## Conclusion

Immediate laparoscopic pyeloplasty for the infant with severe ureteropelvic junction obstruction is effective, and it can accelerate the recovery of renal morphological indices in infants with severe UPJO-induced hydronephrosis.

## Data Availability

The raw data supporting the conclusions of this article will be made available by the authors, without undue reservation.
